# Early Transcriptional Changes in Rabies Virus-Infected Neurons and Their Impact on Neuronal Functions

**DOI:** 10.3389/fmicb.2021.730892

**Published:** 2021-12-13

**Authors:** Seonhee Kim, Florence Larrous, Hugo Varet, Rachel Legendre, Lena Feige, Guillaume Dumas, Rebecca Matsas, Georgia Kouroupi, Regis Grailhe, Hervé Bourhy

**Affiliations:** ^1^Technology Development Platform, Institut Pasteur Korea, Seongnam, South Korea; ^2^Institut Pasteur, Université de Paris, Lyssavirus Epidemiology and Neuropathology Unit, Paris, France; ^3^Université de Paris, Doctoral School Bio Sorbonne Paris Cité, Paris, France; ^4^Institut Pasteur, Université de Paris, Hub de Bioinformatique et Biostatistique, Département Biologie Computationnelle, Paris, France; ^5^Institut Pasteur, Université de Paris, Plate-Forme Technologique Biomics, Centre de Ressources et Recherches Technologiques (C2RT), Paris, France; ^6^Department of Psychiatry, CHU Sainte-Justine Research Center, University of Montreal, Montreal, QC, Canada; ^7^Mila, Quebec Artificial Intelligence Institute, University of Montreal, Montreal, QC, Canada; ^8^Laboratory of Cellular and Molecular Neurobiology-Stem Cells, Department of Neurobiology, Hellenic Pasteur Institute, Athens, Greece

**Keywords:** rabies virus, transcriptome, early post-infection, matrix protein, neuronal dysfunction, calcium imaging

## Abstract

Rabies is a zoonotic disease caused by rabies virus (RABV). As rabies advances, patients develop a variety of severe neurological symptoms that inevitably lead to coma and death. Unlike other neurotropic viruses that can induce symptoms of a similar range, RABV-infected *post-mortem* brains do not show significant signs of inflammation nor the structural damages on neurons. This suggests that the observed neurological symptoms possibly originate from dysfunctions of neurons. However, many aspects of neuronal dysfunctions in the context of RABV infection are only partially understood, and therefore require further investigation. In this study, we used differentiated neurons to characterize the RABV-induced transcriptomic changes at the early time-points of infection. We found that the genes modulated in response to the infection are particularly involved in cell cycle, gene expression, immune response, and neuronal function-associated processes. Comparing a wild-type RABV to a mutant virus harboring altered matrix proteins, we found that the RABV matrix protein plays an important role in the early down-regulation of host genes, of which a significant number is involved in neuronal functions. The kinetics of differentially expressed genes (DEGs) are also different between the wild type and mutant virus datasets. The number of modulated genes remained constant upon wild-type RABV infection up to 24 h post-infection, but dramatically increased in the mutant condition. This result suggests that the intact viral matrix protein is important to control the size of host gene modulation. We then examined the signaling pathways previously studied in relation to the innate immune responses against RABV, and found that these pathways contribute to the changes in neuronal function-associated processes. We further examined a set of regulated genes that could impact neuronal functions collectively, and demonstrated in calcium imaging that indeed the spontaneous activity of neurons is influenced by RABV infection. Overall, our findings suggest that neuronal function-associated genes are modulated by RABV early on, potentially through the viral matrix protein-interacting signaling molecules and their downstream pathways.

## Introduction

Rabies is a fatal disease that occurs through the transmission of lyssavirus, mainly by one of the species called rabies virus (RABV) (Fooks et al., 2014, 2017; Hampson et al., 2015). The virus is transmitted from infected animals to humans through broken skin caused by bites or scratches, or on rarer occasions, licking on the mucosa. RABV transmission from dog-mediated exposures is responsible for more than 98% of human rabies cases, leading to approximately 59,000 deaths every year. Rabies is hard to diagnose without a history of exposure since at the initial stage of the disease the symptoms are very similar to that of many other diseases (Hemachudha et al., 2013). However, as rabies advances, more specific symptoms appear which may include insomnia, anxiety, confusion, paralysis, excitation, hallucinations, agitation, difficulty in swallowing, hydrophobia and aerophobia (Jackson, 2014). The fatality rate is close to 100% once these neurological symptoms have manifested, and as to this date, there are no known cures or treatments that can effectively block the advancement of RABV (Dacheux et al., 2011; Rogée et al., 2019).

Human rabies cases do not show prominent pathological features such as neuron degeneration, adaptive immune cell infiltration, aggravated inflammation, or apoptosis during routine histopathological analysis (Adle-Biassette et al., 1996; Jackson, 2014; Davis et al., 2015; Fisher et al., 2018). This is due to the fact that RABV is capable of evading or delaying immune responses and cell death for the duration of replication and spread in the infected body. RABV encodes five viral proteins in its genome, Nucleoprotein (N), Phosphoprotein (P), Matrix protein (M), Glycoprotein (G), and Large RNA polymerase protein (L), all of which actively mediate signaling pathways to subvert innate immune responses. Three of the viral proteins (P, M, and G) have been more extensively studied. The C-terminal domain of P protein is known to bind to STAT1/2, therefore blocks the translocation of the STAT protein complex (Vidy et al., 2005; Wiltzer et al., 2014) and suppresses the expression of antiviral effectors. M protein has been demonstrated to interact with RelAp43, a splicing analog of RelA (Luco et al., 2012; Khalifa et al., 2016; Besson et al., 2017) and intervenes with the activation of IFN-β and TNF-α. Conversely, a mutant of RABV, in which four critical residues were altered on M protein gene to hinder the interaction with RelAp43, showed to induce a stronger inflammatory cytokine response and a lower pathogenesis *in vivo* than the wild type virus (Sonthonnax et al., 2019). G protein also contributes to the replication of the virus by associating with MAST2 and PTPN4, which alters the balance of PI3K-AKT pathway and enables the infected neuron to evade apoptosis (Préhaud et al., 2010; Caillet-Saguy et al., 2015). The two other viral proteins, N (Masatani et al., 2013) and L (Tian et al., 2016) proteins have shown to be involved in the modulation of the host immune response but their mechanisms of action have been less studied. Even though these modulations by RABV may partially explain the lack of major inflammation and neuronal death, and how the network of neurons is relatively well preserved up until the demise of the host, the mechanism of neuronal dysfunction, which is the probable cause of neurological symptoms, remains elusive.

Different studies showed that the virus or virus infection-associated changes in host gene expression could affect neuron activity. Inflammatory cytokines, such as TNF-α, IFN-γ or IL-1β were demonstrated to desensitize neurons to GABA stimulation and subsequently cause hyperexcitability (Clarkson et al., 2017). Increase of inflammatory cytokines in the brain could result in cognitive impairment and seizure as demonstrated in animal models (Nimmervoll et al., 2013; Habbas et al., 2015). Phosphoprotein of Borna disease virus competes with substrate proteins of PKC and decreases the phosphorylation level of PKC downstream proteins, such as SNAP25 and MARCKS. This action directly affects the synaptic vesicle recycling and structural integrity of synapse, and therefore disrupting synaptic plasticity (Bétourné et al., 2018). In addition, there are studies showing hyperpolarization and disturbance of neuronal network synchronicity as a result of RABV infection (Gourmelon et al., 1991; Ceccaldi et al., 1993; Iwata et al., 1999; Fu and Jackson, 2005); This was shown to be partly due to the lower membrane conductance of Na + and possibly reduced functional expression of the corresponding ion channels. However, the changes in membrane potential and the associated neuronal dysfunction could originate from multiple other mechanisms that remain unexplored, and more importantly, their connections to the RABV infection and the altered signaling pathways in neurons have not yet been properly studied.

RNA sequencing has been a crucial tool to understand gene expression kinetics in comparative studies. Here, we analyze the RNA transcriptome of RABV-infected human neurons in the early hours of infection and attempt to identify the origin of RABV-induced neuronal dysfunction, tracking the signaling pathways that are directly affected by the viral modulation.

## Materials and Methods

### Viruses and Reverse Genetics

Two recombinant rabies viruses were generated through reverse genetics from the rabies strain 8743THA (EVAg collection, Ref-SKU: 014V-02106), an isolate collected from the brain of a man who died of rabies after a dog bite in Thailand. In both viruses, E2-Crimson fluorescence gene (Clontech, 632556) was inserted between M and G protein genes of RABV (Besson et al., 2019) to obtain, the wild-type virus, Tha-E2-Crimson (Tha) and an M protein mutant virus, Th4M-E2-Crimson (Th4M). The matrix protein of Th4M virus differs from Tha’s at 4 amino acid positions: R77K, D100A, A104S and M110L. Both viruses were constructed and rescued as already described (Khalifa et al., 2016; Besson et al., 2019). Full-length viral cDNA (2.5 μg) and plasmids N-pTIT (2.5 μg), P-pTIT (1.25 μg), and L-pTIT (1.25 μg) were transfected in 106 BSR T7/5 cells (Buchholz et al., 1999) in 6-well plates. Cells were then passaged every 3 days until 100% of the cells were infected. The supernatant was harvested and titrated on BSR cells to determine the multiplicity of infection (MOI), following a previously described titration method (Hellert et al., 2020).

### Cells

BSR cells (Sato et al., 1977) were cultured at 37°C, 5% CO_2_ in Dulbecco’s minimal essential medium (DMEM) supplemented with 10% calf serum. BSR-T7 cells (Max von Pettenkofer Institute and Gene Center, Munich) (Buchholz et al., 1999) were cultured in Glasgow medium supplemented with 10% calf serum, tryptose phosphate, non-essential amino acids and geneticin. Neurons used in this study were differentiated either from neural progenitor cells (NPCs) derived from human induced pluripotent stem cells (hiPSCs) generated from a healthy subject (Kouroupi et al., 2017) or H9-NSC (H9 human ESC-derived neural stem cell, Invitrogen, Cat. No. N7800-100).

### Differentiation and Infection of Neurons

hiPSC-derived NPCs were maintained only up to passage number 10 for the usage of neuronal differentiation, in DMEM/F12 medium (DMEM/F12/Glutamax (Gibco 10565-018), supplemented with N-2 1X (Gibco 17502-048), B27 1X (without vitamin A, Gibco 12587010), Penicillin/Streptomycin 1% (Gibco, 15140-122), HEPES 20 mM (Gibco, 15630080), MEM Non-essential amino acid 1X (Gibco, 11140050), bFGF 20 ng/ml (CHAMEDITECH) to the final concentration) and on Poly-L-Ornithine (Sigma P4957) and Laminin (Sigma L2020)-coated T25 flasks. Medium for maintenance was refreshed every second day. On day 0 of differentiation, the NPCs were dissociated by incubation with Accutase (StemPro, A1110501) for 30 min at 37°C, counted and seeded onto PLO/Laminin-coated 96-well plates or 24-well plates for RNA-Seq and RT-qPCR, or 384-well plates for calcium imaging with 10 μM ROCK inhibitor (Y27632; Tocris 1254). The ROCK inhibitor was withdrawn within 2 h after seeding, and fresh Neurobasal medium for differentiation (Neurobasal (Gibco 21103-049), supplemented with B27 2%, and Glutamax 1X to the final concentration) was added onto the cells. The differentiation medium was 50% refreshed every 2∼3 days for 2 weeks. On day 14 of differentiation, the hiPSC-derived neurons were subjected to RABV infection with a MOI 3 and media was changed after 2 h. Two days post-infection hiPSC-derived neurons were subjected to RNA preparation for RNA-Seq or RT-qPCR, and 5 days post-infection were fixed with 4% paraformaldehyde (Thermo Fisher Scientific, AAJ19943K2) and analyzed by immunocytochemistry or were processed for calcium imaging. H9-NSCs were maintained and differentiated in N2B27 medium on PLO/Laminin-coated flasks. N2B27 medium was 1:1 mixture of N2 and B27 media: N2 medium was composed of DMEM/F12/Glutamax, N-2 1X, MEM Non-essential amino acids 1X, Insulin 5 μg/ml (Sigma I9278-5ML), 2-Mercapoethanol (Sigma M7522) and Pen/Strep 1% to the final concentration. B27 medium was composed of Neurobasal, B27 1X, Glutamax 1X, Pen/Strep 1% to the final concentration. EGF 20 ng/ml (Millipore GF144) and bFGF 20 ng/ml were added to N2B27 medium to complete NSC maintenance medium. For differentiation medium, B27 with vitamin A (Gibco, 17504044) was used instead and the growth factors were withdrawn. BDNF (Peprotech, 450-02) and GDNF (Peprotech, 450-10) were added to the differentiation medium to aid neuron survival at a final concentration of 10 ng/ml each. On day 0 of H9-NSC differentiation, cells were detached with Accutase and seeded in T25 flasks in 20% confluence. Within 2 h after seeding, the medium was replaced by N2B27 differentiation medium and once more the next day. The medium was refreshed every second day until day 12. On day 12, the post-mitotic H9 neurons were gently dissociated from T25 flasks and seeded onto 24-well plates for RT-qPCR or 384-well plates for calcium imaging with ROCK inhibitor. The inhibitor was withdrawn within 2 h, and the differentiation medium was 50% refreshed every 2∼3 days up until day 27. The neurons were subjected to RABV infection as above and subsequently used for calcium imaging and RT-qPCR at 2 days post-infection.

### RNA Preparation, RNA-Seq and Data Processing

Total RNAs from collected cell pellets were prepared using Trizol (Invitrogen, Cat. No. 15596026) and ethanol precipitation. For RNA-Seq, non-infected/infected neurons were collected in biological triplicates at 0, 8, 24, and 40 h post-infection using Accutase and kept as pellets at –80°C until the RNA extraction. RNA preparation followed the Trizol manufacturer’s protocol, with addition of Glycogen 10 μg per sample to increase RNA yield. After that, RNAs were treated with DNase I (Promega, Cat. No. M6101) for 30 min at 37°C followed by second Trizol extraction. The acquired RNAs were mixed with 3 volumes of ethanol and 0.1 volumes of 3M sodium acetate, and kept at –20°C for overnight. Next day, the samples were centrifuged and washed with 75% ethanol twice to achieve RNAs in high purity. Sequencing libraries were constructed from the polyA RNA fraction using Illumina TruSeq Stranded mRNA library kit and were sequenced on the Illumina NovaSeq 6000 targeting 12 giga-base reads per sample. After that, raw data was subjected to data cleaning to remove adapter sequences and low-quality sequences using cutadapt version 1.11 (Martin, 2011). Only the sequences greater than 25 nucleotides (nt) in length were considered for further analysis. Mappings to reference genomes were conducted using STAR version 2.5.0a (Dobin et al., 2013) for human DNAs (reference genome: Human (GRCh38) from ENSEMBL version 98) and Bowtie version 1.2.2 (Langmead et al., 2009) for viral DNAs (reference genome: rabies virus genome from NCBI) with default parameters. Following this, genes were counted using featureCounts version 1.4.6-p3 (Liao et al., 2014) from the Subreads package (parameters: -t gene -g gene_id -s 2 -p). An estimated expression level (or read count) for each annotated gene was achieved, and statistical analyses followed using R packages, including DESeq2 (Love et al., 2014). The statistical analysis process includes data normalization, graphical exploration of raw and normalized data, test for differential expression for each feature between the conditions, raw *P*-value adjustment and export of lists of features with significant differential expression between the conditions. The genes with adjusted *P*-value less than 0.05 were considered as differentially expressed compared to non-infected condition. The datasets generated and analyzed for this study can be found in NCBI’s Gene Expression Omnibus (accession number GSE178583).

### Geneset Enrichment Analysis With Gene Ontology and Kyoto Encyclopedia of Genes and Genomes Databases, Keywords Filtering, and Upstream Analysis

**FIGURE 1:**
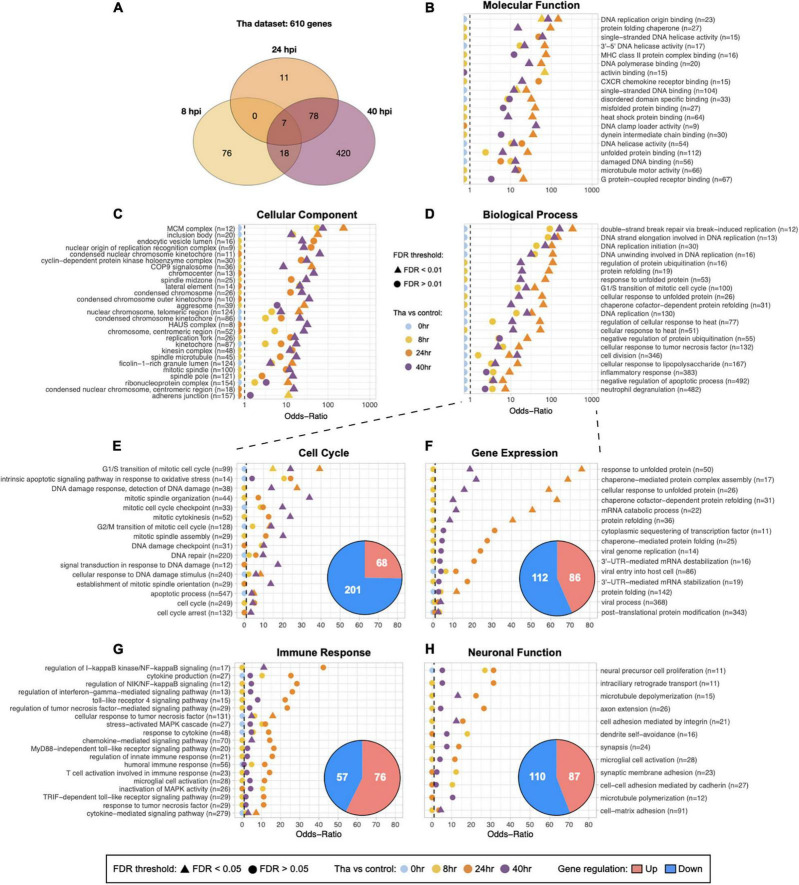
Global and specified GO analysis of DEGs upon Tha infection. **(A)** Venn diagram for the number of genes that were significantly regulated at one time-point or more. **(B–D)** Subsets of categorized GO terms identified with Tha-induced DEGs were presented in each panel. The GO terms for Molecular Function **(B)** and Cellular Component **(C)** were selected by False Discovery Rate (FDR) < 0.01, size of geneset from 20 to 200, or the number of DEGs > 5 for display. Twenty GO terms from Biological Process were selected among the genesets that scored high Odds-ratios on 24 and 40 h time-points, and displayed on **(D)**. Full list of the selected GO terms for each categories is provided in Supplementary Table 4. Biological Process was further filtered into 4 groups, Cell Cycle-related GO terms **(E)**, Gene Expression **(F)**, Immune Response **(G)**, Neuronal Function **(H)** and the number of up or down-regulated genes indicated in pie charts. The GO terms that have Odds-ratios higher than 10, or FDR smaller than 0.05 on at least one time-point are displayed in panels, and the full list the specified GO terms is provided in Supplementary Table 2. The numbers in parentheses indicate the size of geneset for each GO term.

Over-Representation Analysis was performed to generate lists of GO/Kyoto Encyclopedia of Genes and Genomes (KEGG) terms that are over-represented by the differentially expressed genes (DEGs): Enrichment scores for genesets were calculated based on log_2_ fold-change rankings of DEGs. *P*-value was used to calculate false discovery rate (FDR; adjusted *P*-value) in a given geneset, that is a probability of the geneset with an enrichment score to be falsely over-presented. Odds-ratio of each geneset was calculated by the following formula: Odds-ratio = (DEG in geneset/non-DEG in geneset)/(DEG outside of geneset/non-DEG outside of geneset). In order to highlight the relevant ontologies and pathways to the study, subsets of Biological Process (GO) or KEGG Pathways were created by detecting the same set of keywords (stringr package, R Studio). For instance, the keywords we used to produce “GO_interest” or “KEGG_interest” included “signal,” “cascade,” “cytokine,” “receptor,” “transport,” “neuron” and 24 more, and the produced lists of subsets were provided on Supplementary Table 1. For GO, 767 terms were selected out of 2681, and 72 KEGG terms out of 323 from this filtering. Relevant keyword filtering to detect cell cycle, gene expression, immune response or neuronal function-associated GO terms was applied on Figures 1E–H and the generated lists of GO terms were provided on Supplementary Table 2. For upstream analysis and DEG grouping per pathway, KEGG pathway database, TRANSFEC (geneXpression), GeneCards (GeneHancer Regulatory Elements), STRING, and bibliography search were used as references.

### RT-qPCR

For the selected and grouped DEGs, we confirmed the gene regulation at 40 h post-infection (hiPSC-derived neurons) or 48 h post-infection (H9-NSC neurons) by RT-qPCR. Total RNAs were prepared from Trizol extraction and ethanol precipitation, and RT-qPCRs were carried out using SuperScript IV VILO master mix with ezDNase (Invitrogen, Cat. No. 11766050) and QuantiTect SYBR Green PCR kit (Qiagen, Cat. No. 204143) following manufacturer’s protocols. Specific primers for each DEG were selected from QuantiTect Primer Assay (Qiagen, Cat. No. 249900), and listed in Supplementary Table 3. Viral RNA level was assessed by N protein-specific primers (N1130: 5′-CTGACGTAGCACTGGCAGAC-3′; N1247: 5′-AGTCGACCTCCGTTCATCAT-3′), and human GAPDH (forward 5′-GAAGGTGAAGGTCGGAGT-3′; reverse 5′-GGTCATGAGTCTTCCACGAT-3′) was measured as a reference gene for each sample. One-way ANOVA was used to validate the significant differences between conditions.

### Spontaneous Activity Recording With Calcium Imaging

At the end-point of RABV infection, differentiated human neurons were incubated with 3 μM Fluo-4 AM calcium indicator (Invitrogen, Cat. No. F14201) at 37°C for 40 min in 50% BrainPhys Neuronal Medium (STEMCELL, Cat. No. 05790) and 50% of differentiation medium. BrainPhys medium (Bardy and Gage, 2015) was supplemented with 2% B27, and used as loading and reading medium in this study. Subsequently, the cells were gently washed by 70% medium change for 4 times to remove excessive Fluo-4 probe and subjected to recording at 1.72 Hz for 4.5 min with 10X Air objective lens at 37°C (Operetta system, PerkinElmer). From the collected TIFF images, information on the calcium oscillations of each detected soma (cell body of a neuron) was extracted, using FIJI macro and CalBlitz-incorporated Python scripts (open source)^1^ : briefly, motion correction was applied on each image to find the common area between frames of the time-lapse acquisition (Giovannucci et al., 2019). Only the area commonly detected on all frames was subjected to the next analysis step. From the motion-corrected images, regions for the individual somas were segregated by applying binary definition (cell/background) and watershed algorithm. Subsequently, the relative intensity variation ΔF/F for each soma was calculated from Fluo-4 intensity information of the images, by dividing intensity of the individual soma by the average intensity of all frames. After that, the raw ΔF/F trace was transformed from non-linear to linear trend by correlating with a mathematical model of neuron impulse. From the transformed trace of ΔF/F, we extracted the frequency and amplitude information of calcium peaks, by setting the threshold to 2% of ΔF/F. The number of calcium peaks was presented in individual value graphs (Prism 9). Further description of calcium imaging analysis and exemplary intensity trace can be found in “Results” section and Supplementary Materials.

### Immunocytochemistry and Image Analysis

Non-infected or infected human neurons differentiated from neural progenitor cells were fixed with 4% paraformaldehyde for 30 min at room temperature, and washed with PBS 3 times. Neurons were subsequently incubated with a primary antibody against TUBB3 (1:1,000; BioLegend, Cat. No. MMS-435P), diluted in PBS with 5% normal goat serum (Gibco, PCN5000) for 2 h at 37°C, followed by incubation with the appropriate secondary antibody conjugated to AlexaFluor 488 (Thermo Fisher Scientific, Cat. No. A28175) for at least 1 h at room temperature. Nuclei were counterstained with Hoechst (1:1,000). For RABV N protein detection, Anti-Rabies Nucleocapsid Conjugate (Bio-Rad, Cat. No. 3572112) was used according to the manufacturer’s protocol. Following the staining, images were obtained from Operetta or Opera Phenix (PerkinElmer) with 20X Air objective lens and analyzed in Columbus Image Data Management System (PerkinElmer).

## Results

### Temporal Dynamic Expression of Significantly Selected Genes in Wild-Type Rabies Virus-Infected Neurons

To understand the transcriptional changes at early time points of RABV infection and distinguish the modulated processes or pathways that are potentially involved in neuronal dysfunction, we infected 2 weeks-differentiated neurons with wild-type RABV (Tha virus) and performed RNA-Seq: at 8, 24, 40 h post-infection (Figure 1 and Supplementary Figures 1A,B) in biological triplicates. At each time-point, infected samples were compared to the non-infected one to detect the DEGs.

Overall, the datasets showed strong correlations between experimental conditions and gene expression as described in Supplementary Figure 1C. The replicates are clustered according to non-infected/infected conditions and to the time-points, which demonstrates the genuine effects of RABV infection on the transcriptome changes. As the post-infection time-point-dependent changes in gene expression was an intriguing aspect of the datasets, we ran the DEG dataset that were obtained from comparison between Tha virus-infected and non-infected conditions (Tha dataset) through Gene Set Enrichment Analysis (GSEA) pipeline. In total, 610 genes are significantly modulated in Tha dataset (Figure 1A), with 101 and 96 genes at 8 and 24 h, respectively, and then a larger group of genes (516) at 40 h post-infection (Table 1). There are only 7 DEGs detected as common between 8 and 24 h datasets, and 81% of 8 h DEGs are in fact down-regulated. The DEGs are involved in diverse range of biological compartments and functions, as the Gene Ontology (GO) analysis laid out on Figures 1B–D and Supplementary Table 4. The number of GO genesets of which the DEGs are involved in exponentially increases over time, and particular genesets have higher Odds-Ratio on 24 h post-infection than on 40 h (Figure 1D). At 8 and 24 h post-infection, DEGs are detected in 672 and 596 terms in Biological Process, respectively, and then in 1,535 terms at 40 h (Supplementary Table 4). The GO terms that are associated with each time-point also varies. Between 8 and 24 h GO terms, there are only 237 terms in common, in contrast to the comparison of 24 and 40 h which shows 585 common terms in Biological Process (Supplementary Table 4). Combined together, these facts strongly suggest that wild-type RABV impacts the RNA transcription of the infected neurons differently at the earlier stage of infection compared to the later stages.

**TABLE 1:** DEG regulation in Tha and Th4M datasets. Number of up- and down-regulated genes are marked in parentheses.

DEGs in dataset	8 h (up/down)	24 h (up/down)	40 h (up/down)
Tha	101 (19/82)	96 (55/41)	523 (210/313)
Th4M	18 (7/11)	419 (117/302)	624 (364/261)
Common genes	14 (5/9)	51 (32/19)	372 (145/227)

### Neuronal Function-Related Processes Are Regulated Before Immune Response Processes

To understand which processes are regulated early on during the RABV infection and how they evolve over time, further analysis focusing on GO terms in Biological Process was performed. We filtered the GO terms using a keyword detection function in R dplyr package, and assembled 4 groups of GO terms that are relevant to the virus propagation and neuronal dysfunction: Cell Cycle (cell division, DNA damage and repair, apoptosis), Gene Expression (RNA transcription, protein translation and folding, vesicle transportation), Immune Response (cytokines and interferon, innate immune-response-related pathways), and Neuronal Function (neuron projection and synapse formation, extracellular matrix, ion balance, membrane potential) (Figures 1E–H). Up- and down-regulated genes were distributed differently in each group, for instance in the group Cell Cycle (Figure 1E), the down-regulated genes compose 75% of DEGs of the group. DEGs from this group are connected to 120 GO terms (Supplementary Tables 2, 4). Further, the Odds-ratios of Cell Cycle GO terms gradually increase over time until 40 h post-infection, differently from the GO terms of other three groups, and most of these Cell Cycle GO terms have FDR smaller than 0.05 at the last time-point. Illustrating these points, Cell Cycle GO terms with the most significant Odds-ratios such as “G1/S transition of mitotic cell cycle” (27 out of 30 DEGs down-regulated), “mitotic spindle organization” (16 out of 17) and “mitotic cytokinesis” (16 out of 16) showed a higher ratio of down-regulated DEGs at 40 h. In comparison, the group Gene Expression (Figure 1F) presents GO terms with higher Odds-ratios at 24 h compared to 40 h post-infection, implicating that the relevant biological processes are significantly regulated by RABV at 24 h. It shows 198 DEG involved in 87 GO terms (Supplementary Tables 2, 4), related to mRNA stabilization, protein folding, or sequestering transcription factor with significant Odds-ratio. Similar kinetics have been observed on the group Immune Response (Figure 1G). Innate immune response-associated 133 genes are involved in GO terms related to cytokine production, regulation of NF-kB signaling, or response to TNF among others. Close to 70% of DEGs are up-regulated in this group, including *NFKB1, NFKB2, RELB*, and *NFKBIA*, all detected from 24 to 40 h. On the other hand, the genes involved in the group Neuronal Function (Figure 1F) are detected from earlier on, among 212 GO terms 87 of them (41%) contain DEGs that are regulated at 8 h post-infection. Many of genes in this group such as *CCL2, TNFAIP3, NFKB2, MEF2C*, and others are implicated in both major signaling pathways involved in immune response and also in important processes for neuronal functions, which include dendritic morphology, synaptic membrane adhesion, neuronal differentiation, neurotransmitter secretion and more (Supplementary Table 4).

### Mutated M Protein Delays the Suppression of Gene Expression-Associated Processes but Does Not Impact the Rabies Virus Control Over Host Immune Response

**FIGURE 2:**
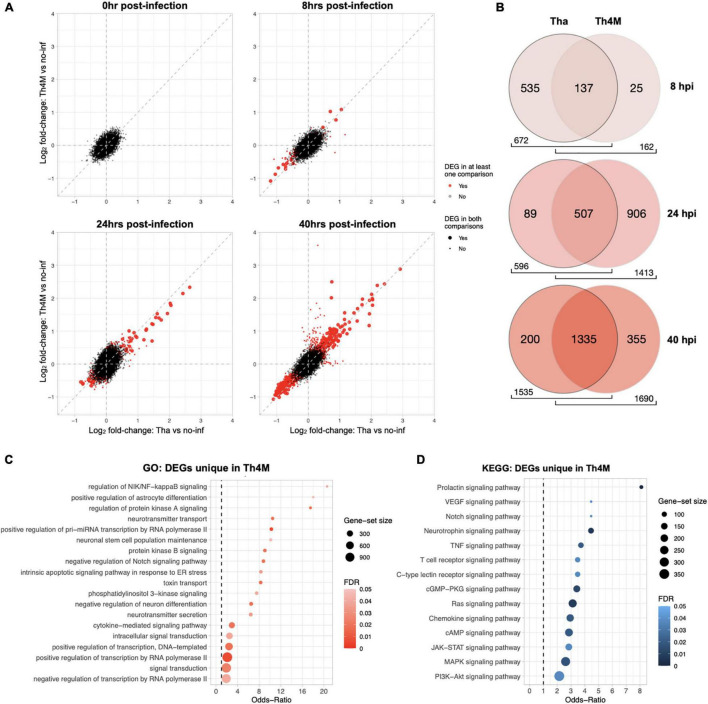
Comparison of Tha and Th4M dataset. **(A)** From 0 to 40 hpi, DEGs (in red) were progressively selected more at the later time points. The common DEGs between two datasets were marked with a larger dot in red, and DEGs unique to one dataset with a smaller dot in red. The exact number of DEGs can be referred to Table 1. **(B)** Th4M dataset shares the majority of associated GO terms (Biological Process) with Tha. As the number of DEGs increases over time, the shared GO terms take the majority of ontology proportion at each dataset. At 8 hpi, Th4M shares 85% of its GO terms with Tha, and at 24 hpi, Tha shares 85% of GO terms with Th4M due to their lower number of DEGs compare to the opponent. At 40 hpi, both datasets share over 79% of GO terms with the other. **(C)** The list of DEGs from all time-points that are only detected in Th4M dataset was crosschecked with the relevant GO term subset. Both the GO subset and DEG crosschecks are provided in Supplementary Table 6. **(D)** Crosscheck of unique DEGs in Th4M dataset with the relevant KEGG pathway subset.

Since the failure of autonomic nerve system is postulated as the main cause of RABV-associated death, we used a mutant RABV (Th4M virus; four point mutations on M protein at amino acid positions 77, 100, 104, and 110) that showed better longevities and delays of neurological symptoms in infected mice (Khalifa et al., 2016; Sonthonnax et al., 2019) to infect differentiated neurons and performed a RNA-Seq analysis. As we mentioned above, M protein from Th4M virus lacks the affinity for the NF-κB signaling protein RelAp43 and therefore allows the induction of IFN-β, TNF-α and other pro-inflammatory cytokines upon infection (Khalifa et al., 2016; Besson et al., 2017). We then undertook a comparative ontology analysis on the RNA-Seq datasets, of Tha and Th4M viruses (Figure 2 and Supplementary Figure 2). As expected, DEGs are regulated differently by two viruses at early time-points post-infection. As described on Table 1 and Figures 2A,B, both datasets show eventual increase of the number of DEGs at 40 h post-infection, but the progression of DEGs at earlier time-points greatly differs between the two datasets.

At 8 h post-infection, Tha dataset shows a modulation of 101 genes, while Th4M shows only 18 DEGs at the same time-point (Table 1). Among these 18 DEG, 14 of them are also observed in Tha dataset at the same time-point, and only 4 genes (*ACSBG1, SFRP4, HPCAL4*, and *GLRA1*) are unique to Th4M dataset. The DEGs in Tha dataset at 8 h are associated with 672 Biological Process GO terms compared to 162 terms of Th4M (Figure 2B), but 85% of the Th4M GO terms (137 terms) are shared with Tha dataset at this time-point. Among the 87 DEGs uniquely detected on Tha dataset at 8 h post-infection, 73 DEGs are down-regulated genes associated with DNA replication and repair, kinetochore assembly, response to redox state (associated with 447 GO terms; examples have Odds-ratio >20; Supplementary Table 4), and 14 DEGS are up-regulated genes related to phospholipid homeostasis, mRNA catabolic process, cation ion homeostasis among others (79 GO terms; Odds-ratio >20; Supplementary Table 4).

At 24 h post-infection, Tha dataset maintains a similar number of DEGs (*n* = 95) to that of 8 h post-infection, with 55 up-regulated and 41 down-regulated genes (Table 1). In contrast, Th4M dataset at 24 h shows a dramatic increase of the number of DEGs compared to 8 h, 419 genes with 117 up-regulated and 302 down-regulated. To investigate the potential delays in the transcriptional modulation by Th4M virus, we analyzed the distribution of DEGs and GO terms at different time-points of two datasets. We found that only 4 DEGs are common between Tha 8 h and Th4M 24 h datasets, and 19 DEGs between Tha 8 h and Th4M 40 h datasets. However, the overview of GO term distribution was different from that of DEGs. Sixty-seven percent of Tha 8 h GO terms appears on Th4M dataset at 24 h, and 80% of Tha 8 h GO terms on Th4M 40 h dataset (Supplementary Tables 4, 5). This could mean that even though the transcriptome profiles of Tha and Th4M datasets are distinct, the DEGs of both datasets are involved in similar biological processes, at earlier time-points in the case of Tha compared to Th4M.

For the GO terms detected at 24 h post-infection, 596 terms are associated with Tha dataset and 1413 terms with Th4M (Figure 2B), which is 2.4 times more than that of Tha dataset at the same time-point. We also found that amongst the terms detected with Th4M DEGs at 24 h, 507 terms are shared with Tha 24 h and then an even higher number of terms, 1006, with Tha 40 h dataset. This involves GO terms such as regulation of NF-kappaB signaling (GO:1901222), intrinsic apoptotic signaling pathway in response to ER stress (GO:0070059), cytokine-mediated signaling pathway (GO:0019221), and KEGG terms including TNF signaling (hsa04668), JAK-STAT signaling (hsa04630), MAPK signaling (hsa04010) and PI3K-AKT signaling (hsa04151), as further detailed on Figures 2C,D. Interestingly, most of the immune response-related processes from Th4M dataset overlap with that of Tha dataset, since the numbers of DEGs that are involved in the relevant pathways are very close between the two datasets. At 24 h post-infection, Tha and Th4M datasets show 24 and 18 DEGs associated with Immune response, respectively (Supplementary Tables 4, 5). Commonly, 15 DEGs including *NFKB1, NFKB2, NFKBIA, RELB*, and *TNFAIP3* are detected on both datasets at this time-point, and are associated with pathways that are known to be modulated by RABV: NF-kB, TNF, MAPK, JAK-STAT, PI3K-AKT, IL-17, TLR, NLR, RLR or chemokine pathways (Gerlier and Lyles, 2011; Wiltzer et al., 2014; Caillet-Saguy et al., 2015; Fooks et al., 2017; Besson et al., 2019).

At 40 h post-infection, the two datasets show a relatively similar effect on biological processes, as 1,335 GO terms (87% of Tha and 79% of Th4M terms) (Figure 2B) are commonly detected in both datasets and 372 genes are shared between them (Table 1).

To conclude, Tha and Th4M datasets display different dynamics of gene regulation in differentiated neurons especially at early time-points, but they become similar at 40 h post-infection when the feedback response from host cells becomes more prominent. Overall, Tha virus secures more control over the gene modulation than Th4M at the early hours of infection, which emphasizes the role of M protein in the early regulation of host gene expression.

### Rabies Virus-Associated Pathways Are Involved in Neuronal Differentiation, Synaptic Activity and Morphological Changes in Tha and Th4M Datasets

**FIGURE 3:**
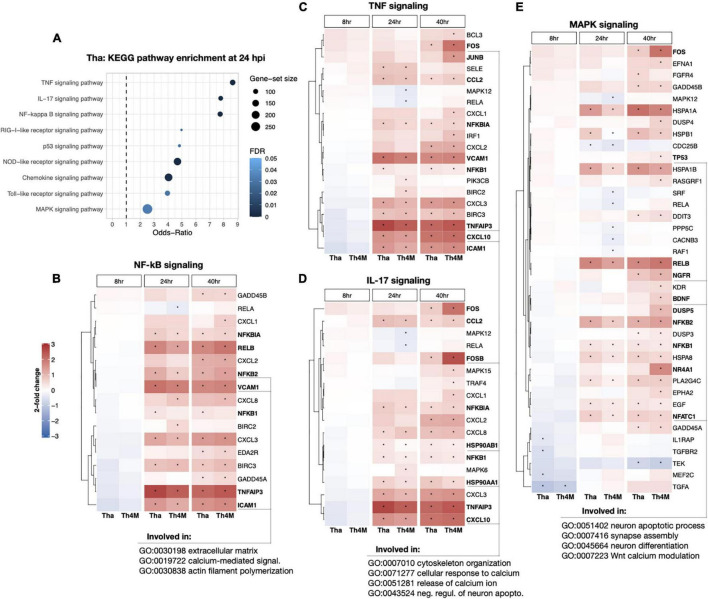
DEG heatmaps of RABV-associated KEGG pathways and the connection to the neuronal function. **(A)** The relevant KEGG subset was crosschecked with DEGs from 24 hpi Tha dataset, highlighting multiple pathways that are important to cell cycle, differentiation, signal transduction, and immune response among others. **(B)** DEGs involved in NF-kB signaling (hsa04064) and the expression level indicated in log_2_-fold changes. The time-points of significant selection were marked with black dot. Examples of DEGs and their association with neuronal function-GO terms are underlined at the base of figure. **(C)** DEGs detected in TNF signaling (hsa04668). **(D)** DEGs in IL-17 signaling (hsa04657). **(E)** DEGs detected in MAPK signaling (hsa04010). DEGs validated in RT-qPCR for their expression at 40 hpi (displayed on Figure 4) are marked in bold font.

To understand which genes in the aforementioned RABV-associated pathways could directly impact neuronal functions or related processes, we investigated relevant DEGs identified in the KEGG pathways. At 24 h post-infection, TNF, IL-17 and NF-kB pathways scored relatively high Odds-Ratios compared to other gene sets (Figure 3A). The DEGs detected in these pathways are mainly up-regulated in both Tha and Th4M datasets (Figures 3B–D), and many of these genes are induced at 24 h post-infection and maintained a high expression level until 40 h post-infection. These pathways are associated through their component genes to biological processes such as extracellular matrix organization (GO:0030198), calcium-mediated signaling (GO:0019722), cytoskeleton organization (GO:0007010), and negative regulation of neuronal apoptosis (GO: 0043524). In comparison, MAPK signaling pathway on Figure 3E shows more down-regulated DEGs than the previously mentioned pathways, notably *IL1RAP* (8 h on Tha; involved in inflammatory response, regulation of pre-/post-synaptic density assembly, positive regulation of NF-kB activity), *CACNB3* (24 h on Th4M; involved in membrane depolarization), *TEK* (40 h on Tha and Th4M; involved in negative regulation of apoptotic process, negative regulation of inflammatory response, positive regulation of PI3K activity) as indicated on Supplementary Tables 4, 5. MAPK pathway is also associated through the subset genes to neuronal function-related biological processes such as synapse assembly (GO:007416), neuron differentiation (GO:0045664), and Wnt calcium modulating pathway (GO:0007223). As presented in the heatmaps, the overall DEG expression ranges from –1.2 to 3 log_2_-fold changes compared to non-infected conditions at each time point. This highlights the stealthy nature of RABV-induced modulation over gene expression in differentiated neurons and relatively mild feedback from the host defense mechanism during the first replication cycle of RABV.

**FIGURE 4:**
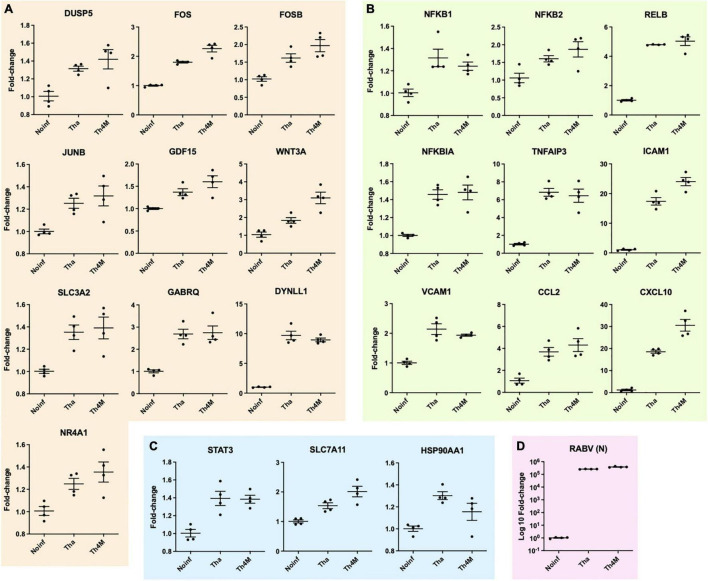
Validation of neuronal function-related DEGs with RT-qPCR at Tha- and Th4M-infected condition and grouping in the associated pathways. **(A)** DEGs associated with MAPK signaling. DUSP5, FOS, FOSB, JUNB are detected within KEGG MAPK signaling geneset, and the association of the remaining group was identified using different tools for upstream search (refer to text). **(B)** DEGs detected directly in KEGG NF-kB signaling geneset are NFKB1, NFKB2, RELB, NFKBIA, TNFAIP3, ICAM1 and VCAM1. **(C)** STAT3 was directly detected in JAK-STAT KEGG geneset, and the associations of SLC7A11 and HSP90AA1 were identified through upstream search. **(D)** RABV RNA level was detected in RT-qPCR using N protein primers. All comparisons between experimental conditions showed statistical significance (one-way ANOVA, *P*-value < 0.05 or lower).

The up- or down-regulated DEGs of RABV-associated pathways impact neuronal function-related processes through signaling transduction network. We have identified several direct and indirect connections from the signaling transduction-related DEGs to the neuronal function-associated DEGs through upstream search using KEGG pathway database, TRANSFEC (geneXpression), GeneCards (GeneHancer Regulatory Elements), STRING and bibliography search. The examples of MAPK, NF-kB, and JAK-STAT pathway DEGs that are associated with neuronal functions were validated with RT-qPCR and assembled on Figure 4. *DUSP5* on Figure 4A was previously studied for its contribution to the RABV replication (Besson et al., 2019), and plays an important role in de-phosphorylation of active ERK and controlling neurite outgrowth and postsynaptic density (Kim et al., 2015; Lacar et al., 2016; Kidger et al., 2017), along with other DUSP proteins. In our results, *DUSP5* is detected as a DEG in Th4M dataset, but shows increased fold-changes in both Tha and Th4M RT-qPCR validations (Figure 4A and Supplementary Figure 3). Other MAPK pathway downstream elements *FOS, FOSB* and *JUNB* are up-regulated to varying degrees under Tha and T4M infection, which could then directly act as transcription factors for GDF15 [increases Glutamate release through up-regulation of voltage-gated calcium channel (Liu et al., 2016)], SLC3A2 [forms cystine/glutamate transporter with SLC7A11 and regulates glutamate uptake (Bhutia and Ganapathy, 2016)], NR4A1 [NGF nuclear receptor, associated with dendritic spine attrition and cognitive impairment (Jeanneteau et al., 2018)] and GABRQ [extrasynaptic GABA receptor subunit theta (Fatemi et al., 2013)]. As part of GABAA receptor, GABRQ localizes on extrasynaptic membrane of neuron and contributes to GABA-mediated chloride ion fluxes and inhibitory regulation of action potential. All these DEGs have been confirmed to increase under Tha and Th4M infections in both RNA-Seq and RT-qPCR validation. WNT mediates neuronal circuit formation and could be up-regulated by MAPK pathway modulation through the upstream transcription factor CDX2 (De, 2011), which is validated by RT-qPCR. DYNLL1 (LC8) is an important protein involved in the translocation of RABV particles, but also in DNA damage response pathway and anti-apoptotic activity by interacting with TP53BP1, BIM and BMF (Fooks et al., 2017; He et al., 2018; West et al., 2019). *DYNLL1* is up-regulated in our RNA-Seq and RT-qPCR experiments, for both datasets (Figure 4A and Supplementary Figure 3). In Figure 4B, selected DEGs from canonical and non-canonical NF-kB pathways and the possible downstream genes that are up-regulated are presented. NFKB1, NFKB2, RELB, NFKBIA are involved in host immunity and oncogenesis, as well as in synaptic plasticity, communication between synapse and nucleus, response to membrane potential changes and neurite outgrowth through downstream regulation (Kaltschmidt and Kaltschmidt, 2009; Engelmann and Haenold, 2016; Dresselhaus and Meffert, 2019). ICAM1 and VCAM1 could receive signals from both MAPK and NF-kB pathways for expression (Taniguchi and Karin, 2018), and contribute to neuron adhesion, stability of extracellular matrix, and permeability of brain barriers. TNFAIP3 (A20) is involved in negative regulation of inflammatory response and apoptosis, and heterozygosity of this gene causes an exacerbated cognitive impairment under inflammatory conditions (Daems et al., 2020; Martín-Vicente et al., 2020). The primary function of CCL2 and CXCL10 is chemokines for monocytes and macrophages, but also could indirectly cause neuronal dysfunction through the recruited immune cells (Lehmann et al., 2019). The chemokines could also increase intracellular calcium level within neurons and cause hyperpolarization and excitotoxicity (Kodangattil et al., 2012). In Figure 4C, *STAT3* increases under Tha/Th4M infected-condition, and so do the downstream effectors SLC7A11 and HSP90AA1. As mentioned above, SLC7A11 forms Xc- cystine/glutamate anti-transporter, and HSP90AA1 functions as scaffold protein and maintains the integrity of cytoskeletal proteins in neurons as well as induces clearance of abnormally aggregated proteins (Ou et al., 2014). Correlation between RNA-Seq and RT-qPCR results are shown in Supplementary Figure 3, along with expression levels of housekeeping genes that are unaffected by virus infection. The validation of several DEGs are confirmed again in H9-NSC-differentiated neurons, as presented in Supplementary Figure 4.

### Rabies Virus Infection Hinders Spontaneous Activity of Neurons

**FIGURE 5:**
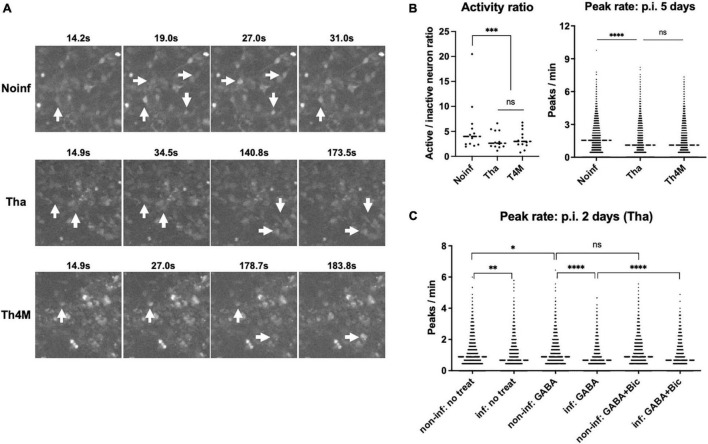
Decreased spontaneous activity in RABV-infected neurons. The hiPSC-derived NPCs were differentiated into neurons for 2 weeks and then infected with Tha/Th4M virus for 5 days at MOI 3. **(A)** Examples of active neurons visualized with Fluo-4 staining. Size of the images represents 1.6% of field of view (150 × 150 pixels out of 1360 × 1024). Individual somas marked with white arrows show the intensity changes over time (active neurons). Video format of the same images is provided in Supplementary Figure 6. **(B)** The active/inactive neuron ratios were counted from 12 imaging sessions of non-infected/infected conditions, and are presented as individual value graph on the left panel. The neurons that had more than one calcium peak detected during the imaging were considered as active. Bartlett’s test evaluated the significant difference between the conditions. On the right panel, the graph represents frequencies of calcium peaks collected from over 5,000 individual somas at each condition. Student’s *t*-test was performed to show the statistically significant difference between conditions. **(C)** H9-NSC-differentiated neurons and the decreased spontaneous activity on 2 days post-infection. The H9-NSCs were differentiated into neurons for 4 weeks and then infected with Tha virus for 2 days at MOI 1. The frequencies of calcium peaks were calculated from over 1,500 individual somas and presented in the graph. The non-infected and infected conditions show a statistical difference in Student’s *t*-test, while the non-infected and infected conditions with GABA treatment (two graphs in the middle) presented stronger difference. GABA_*A*_ receptor antagonist Bicuculline (Bic) partially reversed the effect of GABA and Tha infection, as shown in comparison of GABA/GABA + Bic treatment in infected conditions. Asterisks represent statistical significance; * for *P*-value < 0.05, ^**^ for < 0.005, ^***^ for < 0.0005, and ^****^ for < 0.00005. GABA, Gamma-aminobutric acid.

To understand the impact of RABV infection on neuronal activity, we performed calcium imaging on the NPC-derived neuron cultures. Calcium imaging with Ca^2+^ indicators can reflect the genuine neuronal activity, number of action potentials and the amplitude, correlating with the observed calcium traces (Kirwan et al., 2015). In our experimental setting, neurons expressed a neuronal marker TUBB3 after 2 weeks of differentiation and formed complex network over the desirable size of field of view (Supplementary Figure 5; over 500 somas detectable under 10X lens). The differentiated neurons were then infected with Tha or Th4M virus at MOI 3, same as in the RNA-Seq experiment, and subjected to Fluo-4 staining and calcium imaging on 5 days post-infection (more than 3 days after the last time point of the RNA Seq analysis). The acquired images were processed through Calblitz workflow (Supplementary Figure 6) to extract information on calcium peak frequencies and amplitudes from individual somas. Active neurons, which has more than one calcium peak over the course of imaging, were present in all three experimental conditions (Figure 5A and Supplementary Figure 6). However, Tha- and Th4M-infected neurons exhibited lower neuronal activities compared to the non-infected condition (Figure 5B). The frequency of calcium peaks was significantly higher on non-infected condition than on the Tha-infected condition (right panel), as well as for the ratio of active neurons (left panel). Comparison between Tha- and Th4M-infected neurons was not statistically significant on either activity ratio or the peak rate on 5 days post-infection. Average of amplitude of calcium peaks showed similar heights between non-infected and infected neurons, as presented in exemplary intensity traces in Supplementary Figure 6D. As summarized in graphs on Supplementary Figure 5, the results of calcium imaging did not originate from structural damages such as axonal/dendrite degeneration (neurite count/length) or apoptosis (bleb count). Interestingly, when we reduced the duration of infection to 2 days to observe the effect of Tha virus infection in similar timeline as the RNA-Seq experiment (Figure 5C), the difference of peak rates was less significant between non-infected and infected neurons in comparison to 5 days post-infection. However, the Tha-infected neurons exhibited a sensitivity to the inhibitory neurotransmitter GABA, with the frequency of calcium peak reduced further by the combination of infection and GABA treatment than on other conditions. This may indicate how the neuronal function is affected by the regulation of neurotransmitter-associated genes, such as the GABA receptor subunit GABRQ, under RABV infection.

## Discussion

As rabies advances, signs of autonomic and cognitive neuronal dysfunction become more prominent (Fooks et al., 2017). Many viral encephalitis studies attributed similar neurological symptoms to the strong immune responses from the macrophage/monocytes, neutrophils and adaptive immune cells, infiltrating central nervous system (CNS) or recruited through disrupted blood-brain barriers (Denizot et al., 2012; Ludlow et al., 2016; Carod-Artal, 2018). Excessive immune responses against viruses in the CNS result in neurodegenerative pathologies, such as neuronal death, axon degeneration or demyelination (Tsunoda, 2008; Ramesh et al., 2013; Kennedy, 2015). In contrast, rabies virus has developed strategies to evade immune responses at the site of initial entry as well as in the CNS, and widely spread across the brain even before the onset of symptoms (Jackson, 2006; Hemachudha et al., 2013). In order to understand the early modulations that occur in RABV-infected neurons, we have analyzed the dynamic transcriptome changes of the infected neurons around the first cycle of virus replication in absence of secondary inputs from glial cells, and provided neuronal function-associated genes that could directly be affected by RABV modulation of signaling pathways. The originality of this work lies on the fact that the transcriptome kinetic analysis has been performed from very early time-points of RABV infection up to 40 h post-infection, and that the role of M protein in transcriptome modulation and neuronal dysfunction has been investigated in differentiated human neurons.

### M Protein Regulates Neuronal Function-Associated Genes From Early Time-Points of Infection

At 8 h post-infection, Tha dataset showed faster regulation of host gene expression than Th4M dataset, with the majority of these DEGs being down-regulated (81%). We showed that in Tha dataset, the group Neuronal Function particularly stood out at 8 h compared to the group Cell Cycle, Gene Expression or Immune Response, since 44% of DEGs at 8 h post-infection is associated with neuronal functions. Some of the high Odds-ratios we observe might occur due to the slightly smaller size of gene sets in the group Neuronal Function, or also because the specific gene ontologies are highly influenced by Tha virus infection at 8 h compared to the others. Also, the virus infection may have direct contribution onto this regulation, through immediate early genes (IEGs). IEGs regulation in host cells can be triggered by early event of virus infection—attachment, internalization, intracellular trafficking and uncoating—and activate series of transcription factors and DNA-binding proteins that are best-characterized in Ras/Erk/MAPK-associated signaling pathways (Tullai et al., 2007; Gallo et al., 2018). These genes are induced to maximum level within 1–4 h after stimulation and activate or suppress secondary transcription within few hours. Many of the group Neuronal Function-associated DEGs observed at 8 h (*GRHL3, PDPN, TNFRSF11B, SPP1, COL11A1, PTX3, CRISPLD2, CDH2, EZR, NT5E, SMAD7, MEF2C, CLDN1, RGS4, ANXA1, CEMIP, ARNTL, LEF1, HBEGF, TGFA, WEE1*) have transcription factor binding sites associated with Ras/Erk/MAPK or related pathways upstream (identified by TRANSFEC search), so it is possible that M protein of RABV may interact with Ras/Erk/MAPK signaling pathway proteins and affects the downstream transcription. Another point being addressed regarding the regulation of neuronal function in early time-points is the proportion of down-regulated genes in DEGs. There are 86 processes in the group Neuronal Function at 8 h post-infection, and 64 of them are influenced by down-regulated genes. Compared to the 26 processes of Th4M dataset in the same criteria and the sudden expansion of DEGs at 24 h, the results show how important the role of M protein is in the early repression of host gene expression. Vesicular stomatitis virus (VSV), another rhabdovirus belonging to the Vesiculovirus genus (a genus closely related to that of the lyssavirus genus), also globally suppresses the transcription and translation of the host cells by 4–6 h post-infection, and the M protein of VSV is primarily responsible for this inhibition (Lyles et al., 2013). The scale of host gene expression inhibition induced by RABV M protein seems to be much smaller than that of VSV, as demonstrated in this study. However, if our results suggest that RABV is a milder inducer of host gene expression than VSV, RABV has developed more specific ways to control signaling pathways than general inhibition. This is in line with previous studies showing the crucial role of RABV M protein in the regulation of signaling proteins *in vitro* and *in vivo* (Luco et al., 2012; Khalifa et al., 2016; Besson et al., 2017).

### Size of Differential Expression During the First Round of Virus Replication Is Controlled by M Protein

In Tha dataset, we observed that the control of M protein over the host gene expression was maintained at 24 h post-infection, in contrast to the delayed but drastically increasing number of DEGs on Th4M dataset. It seems that during the first round of virus life cycle (∼24 h post-infection), RABV has the competition between host response and virus replication under its control due to the presence of functional M protein, which implies that the suppression of host gene expression by M protein at early time-points of virus replication maybe significant for keeping the host cells relatively “quiet.” Modulating host gene expression by the virus is not necessarily bound to immune responses here, since Tha and Th4M datasets display similar number of DEGs detected directly in immune response-associated pathways at 24 h (according to KEGG pathway definition) and also similar number of GO terms related to innate immune responses at that time-point (Tha 92 terms, Th4M 105 terms by keyword filtering in Biological Process). This is to a certain degree a discrepancy from the previously reported studies showing stronger immune responses elicited by attenuated RABV (Wang et al., 2005; Koraka et al., 2018). Considering the genetic differences between the virulent RABV strain Tha and its mutant virus Th4M are 4 amino acids, the results we obtained only highlight the specific characters of M protein-derived regulation. Additionally, in our datasets, Th4M dataset has more DEGs that are directly or indirectly involved in gene expression, metabolism, or immune response processes from 24 h post-infection onward, which may affect the kinetics of virus replication as presented in Supplementary Figure 1 and partly cause the delayed neurological symptoms in Th4M-infected mice (Sonthonnax et al., 2019). In the current study, we were able to provide clearer perspectives on the kinetics of the progression of neuronal dysfunction in RABV infection, since we used neuron culture and shorter incubation time. In comparison, Zhang et al. (2016) used intranasally inoculated mice model to study CVS-11 and HEP-Flury transcriptome modification in the brain, but the earliest time point for RNA harvest was 10 days post-infection and the CVS-11 dataset showed over 2,800 DEGs ranging from –2 to 11 log_2_ fold-changes. The analysis showed immune responses and cell death-oriented GO distribution for the CVS-11 dataset, which most likely comes from combination of cell types in the brain and therefore is difficult to interpret regarding the neuronal dysfunction. A study published in 2016 (Jamalkandi et al., 2016) took a systemic approach by meta-analyzing the existing datasets and extracted 7 relevant signaling pathways to RABV pathogenesis (WNT, MAPK, RAS, PI3K/AKT, TLR, JAK-STAT, and NOTCH), which are similar to the KEGG pathways filtered on p.i. 24 h based on higher Odd-Ratios in Figure 3. However, the datasets in this study as well were produced from mouse brain, spinal cord, and microglial cells with varying microarray systems and virus strains, and the time points for the sample collection were unclear. As the primary target for RABV replication, neurons are expressing receptor molecules such as nicotinic acetylcholine receptor (nAChR), neuronal cell adhesion molecule (NCAM), p75 neurotrophin receptor (p75NTR) or metabotropic glutamate receptor subtype 2 (mGluR2) (Zhang et al., 2014; Guo et al., 2019). Neurons are therefore exposed to host transcriptome modulation of RABV earlier than the other types of cells present in the nervous system, astrocyte, microglia or oligodendrocyte. From our study, the changes in neurons at early hours of infection seem crucial to maintain the control of RABV on preexisting host defense system and in particular on neuronal function regulation, as 137 out of 610 DEGs in Tha dataset are involved in neuronal function regulation, and 316 genes out of 969 DEGs for Th4M. Thus, this study provides an important insight on the sceneries of RABV’s first replication cycle in its target domain and how the temporal regulation shifts to eventually affect neuronal functions.

### Immune Response-Associated Pathways That Interact With Rabies Virus Impact Neuronal Functions

Regulation of many neuronal function-associated genes roots from the signaling pathways that are directly affected by RABV infection. One of such pathways is NF-kB signaling pathway. NF-kB pathway is intensively studied for its involvement in host immune responses, but also in relation to neuronal functions as it is localized in synapse for fast signaling transmission. NF-kB is constitutively activated in glutamatergic neurons (e.g., granule cells and pyramidial neurons in hippocampus CA1 and CA3) and responds to the increase of calcium level at the post-synaptic dendritic spine and translocate to nucleus (Kaltschmidt and Kaltschmidt, 2009; Engelmann and Haenold, 2016). Matrix protein of RABV interacts with RelA and more specifically its splicing variant RelAp43. Consequently, this interaction hinders the translocation and re-balances the downstream transcription by NF-kB pathway, including of IFN and TNF (Luco et al., 2012; Khalifa et al., 2016; Besson et al., 2017). RelA mainly partners with NF-kB p50, but also can interact with p52 and translocate to nucleus to act as transcription factor. With external stimulation such as TNF-α, IL1-β, CXCL10 or TGF-β1, or internal stimulation like viral RNA detection by RIG-I or TLRs, the RelA-p50 dimer transcribes inflammatory cytokines and inducible nitric oxide synthases (iNOS), and hinders the virus replication. With RelA stalled by M protein interaction, the downstream of NF-kB pathway weighs more on other transcription factors/co-activators/repressors, such as RelB, c-Rel, p50 and p52, frequently STATs, AP-1, p53 and IRFs in combinations (Taniguchi and Karin, 2018). The shifted transcription spectrum affects wide range of biological processes including neuronal functions, as exemplified by modulated transcription of TNFAIP3, ICAM1 or VCAM1. Also, the TPL2 (MAP3K8)-dependent interaction of M protein to p105 would prevent the positive regulation of MEK1/2 by TPL2 (Besson et al., 2017) and subsequently ERK1/2 or p38 signaling path, which as a result may suppress the expression of inflammatory receptors (Xu et al., 2018). This possibly leads to increasing the survival of neurons, but also alters the signaling pathways that interact with TPL2, such as release and destabilization of A20 binding inhibitor of NF-kB (ABIN2), which subsequently influence A20 (TNFAIP3)-mediated ubiquitination/de-ubiquitination of NF-kB signaling proteins (Webb et al., 2019; Martín-Vicente et al., 2020). Therefore, it is not surprising that Th4M, harboring 4 amino acids changes in the M protein impairing the M protein interaction with RelAp43, is not able to suppress host gene expression at early time-points of virus replication as efficiently as observed with the native virus Tha.

As mentioned before, modification of ERK/MAPK signaling plays important roles in both inflammation- and neuronal function-associated transcriptions. In our study, we have confirmed that several DEGs related to neurotransmitter uptake (SLC3A11, SLC2A11) and postsynaptic activity (GABRQ) are indeed regulated by RABV infection and this could result from the direct interaction between RABV proteins and MAPK pathway components. Of note, host genes of MAPK pathway that are important for virus replication are not necessarily modulated during the RABV infection, since the virus replication-assisting/inhibiting MAPK pathway genes observed in a previous study (Besson et al., 2019) is not seen in our Tha dataset. JAK-STAT pathway could also be significantly modified by RABV infection, as STAT1/2 is efficiently sequestered by interaction with P protein. To a lesser degree in human neurons, P protein also binds to STAT3 (Harrison et al., 2020) and interferes with Gp130 receptor-dependent signaling that are involved in neuroprotection, neurite outgrowth and synapse formation (Lieu et al., 2013; Chen et al., 2016).

## Conclusion

In conclusion, our study shows that the gene expression in neurons is significantly regulated by RABV infection from the early point of virus replication cycle, and M protein of RABV plays important roles in keeping the host gene expressions at bay during this time. RABV infection in neurons especially alters the expression of genes associated with neurotransmitter-gated ion channels/neurotransmitter uptake/morphology and extracellular matrix through RABV-interacting pathways at early hours of infection, which contribute to the eventual decrease of spontaneous activity in the infected neurons.

## Data Availability Statement

The datasets presented in this study can be found in online repository. Details of information is indicated in Materials and Methods.

## Author Contributions

SK, FL, RG, and HB contributed to the design of the study. HV, RL, and GD established analysis pipelines. SK, FL, and LF performed the experiments. SK, FL, HV, and RL analyzed the data. RM and GK provided the materials. SK, FL, and HB wrote the manuscript. All authors contributed to the revision of the manuscript and approved the submitted version.

## Conflict of Interest

The authors declare that the research was conducted in the absence of any commercial or financial relationships that could be construed as a potential conflict of interest.

## Publisher’s Note

All claims expressed in this article are solely those of the authors and do not necessarily represent those of their affiliated organizations, or those of the publisher, the editors and the reviewers. Any product that may be evaluated in this article, or claim that may be made by its manufacturer, is not guaranteed or endorsed by the publisher.

## Footnotes

1
https://github.com/agiovann/CalBlitz
