# Supplementary material for: Early Transcriptional Changes in Rabies Virus-Infected Neurons and Their Impact on Neuronal Functions
Source: Front Microbiol. 2021 Dec 13;12:730892. doi: 10.3389/fmicb.2021.730892 (PMC8713068; doi:10.3389/fmicb.2021.730892)
Supplement: Supplementary file 1 [file Data_Sheet_1.DOCX]

Supplementary Material

# Supplementary Figures


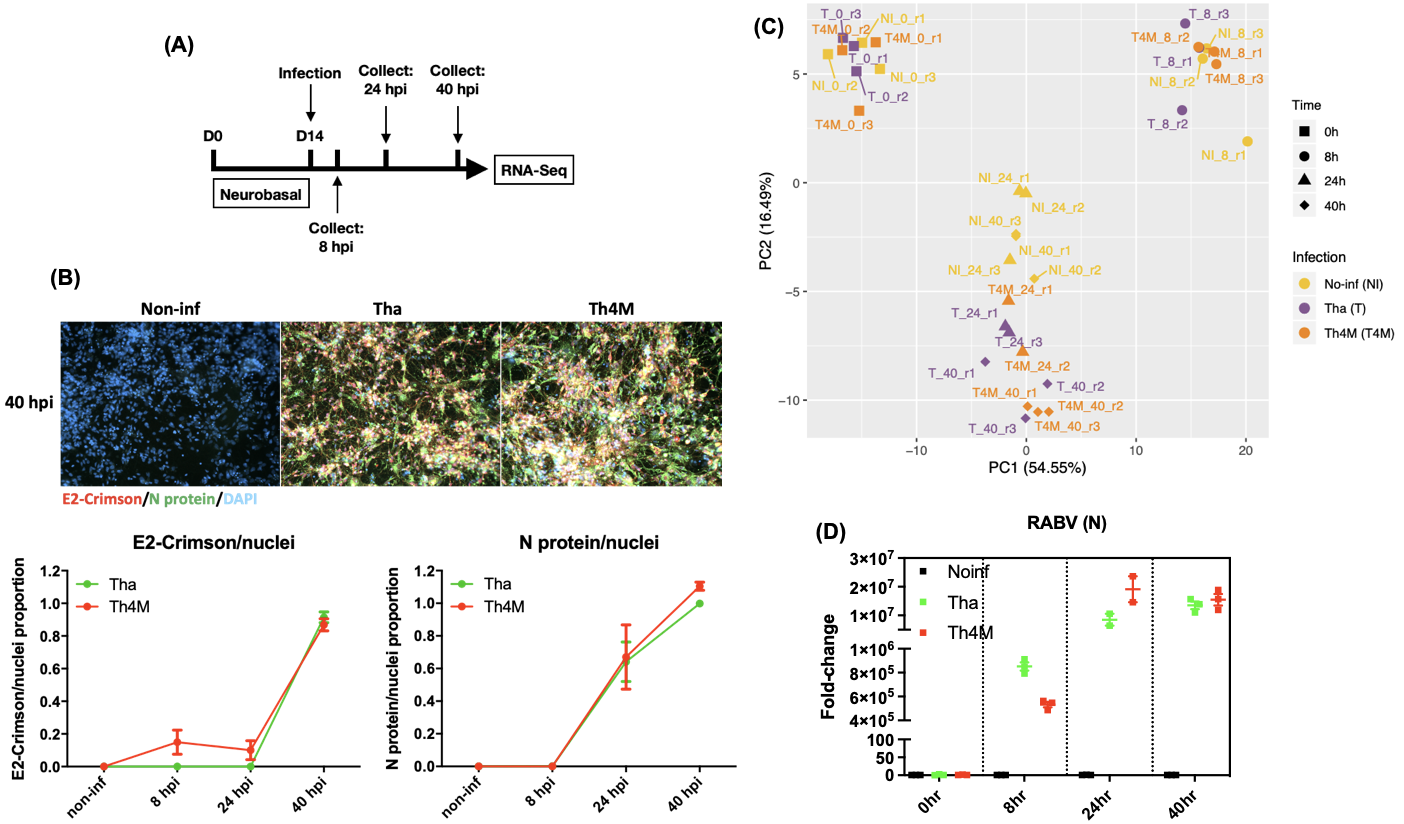


**Supplementary Figure 1.** Experiment design, validation of infection, and clustering of processed RNA-Seq data. (A) VEK4.34 NPCs were differentiated in Neurobasal medium for two weeks, and infected either with Tha or Th4M virus at MOI 3, as biological triplicate. The infected neurons were collected at each time points and the total RNAs were extracted at the same time for RNA-Seq. (B) Images of infected neurons at 40 hpi and the measurement of proportion of E2-Crimson-positive soma (graph on the left) or immunostained soma with N protein antibody (graph on the right) (C) First two components of Principal Component Analysis showing the separation between biological conditions (non-infection, infection, harvest time-point). The PCA is based on the variance-stabilized transformed count matrix that has been adjusted for the replicate effect using the removeBatchEffect function or the limma R package (version 3.44.3). (D) RABV RNA levels at each time-point were measured by RT-qPCR using N protein primers.


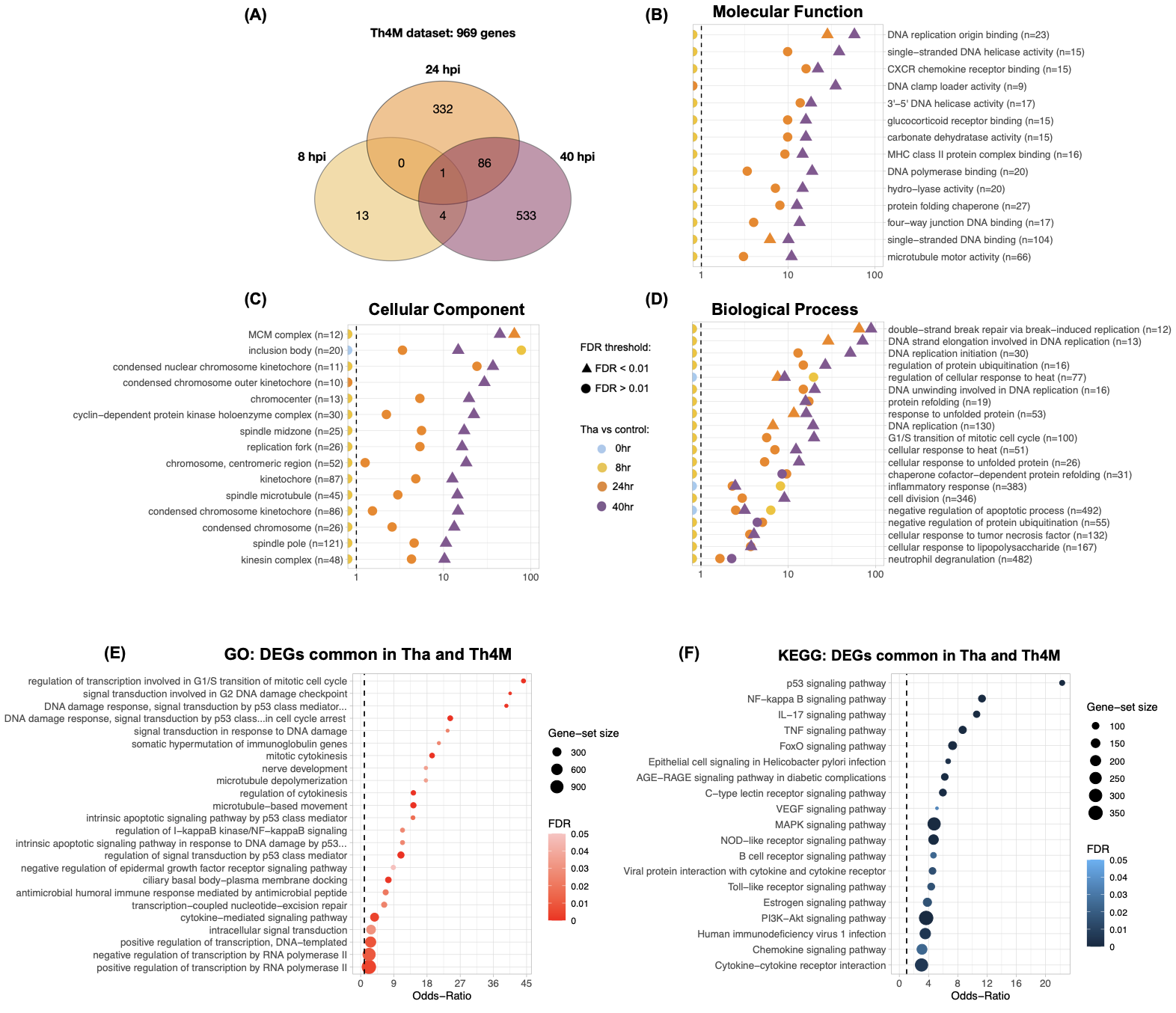


**Supplementary Figure 2.** Global analysis of DEGs upon Th4M infection. (A) Venn diagram for the number of genes that were significantly regulated at one time-point or more. (B-D) Each panel represents the subsets of categorized GO terms. The criteria for GO term selection for panels are same as Figure 1, for Molecular Function (B) and Cellular Component (C) the False Discovery Rate (FDR) < 0.01, size of geneset from 20 to 200, or number of DEGs > 5. Twenty GO terms from Biological Process with high Odds-ratios on 24 and 40hrs were selected and displayed on (D). Full list of the selected GO terms for each categories is provided in Supplementary Table 2. (E) DEGs common in Tha and Th4M datasets were crosschecked with the relevant GO terms subset and displayed. (F) Crosscheck of common DEGs and the relevant KEGG pathway subset.


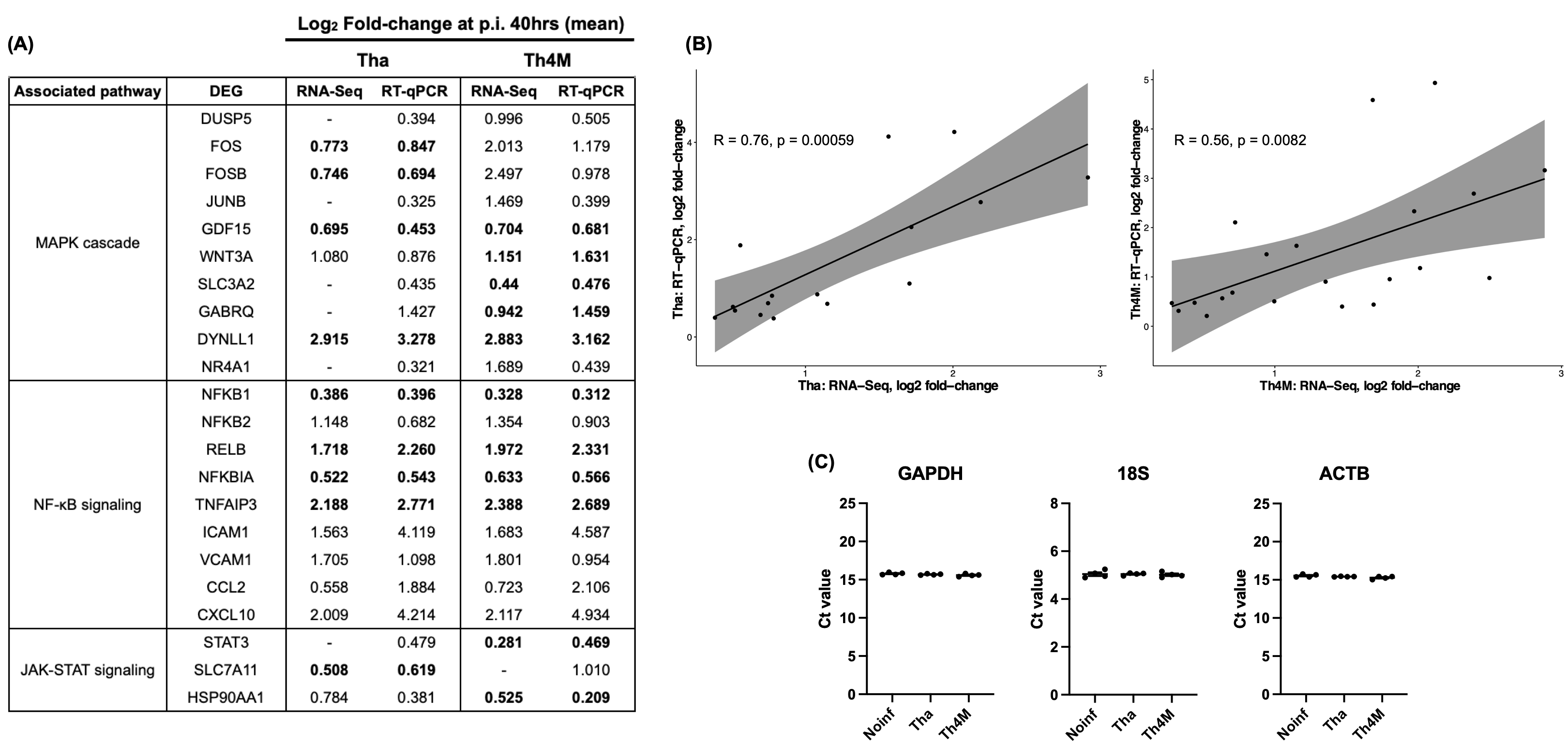


**Supplementary Figure 3.** Correlation between RNA-Seq and RT-qPCR results. The values presented in the table (A) were from Log_2_ fold-changes of DEGs in RNA-Seq datasets (when the gene is not detected as DEG, it is marked with “-”), or RT-qPCR results. The values of RT-qPCR were converted to Log_2_ scale from Figure 4 fold-changes. Values in bold font in the table are detected within the confidence intervals of panel B. (B) Left panel represents the correlation between RNA-Seq and RT-qPCR of Tha dataset, and right panel represents of Th4M dataset. The grey area in the graphs indicates confidence interval. Correlation coefficient R and P value of the correlation are indicated on the panels. (C) Housekeeping genes including GAPDH (reference gene for RT-qPCR) present similar level of expressions between different conditions, unaffected by the virus infection.


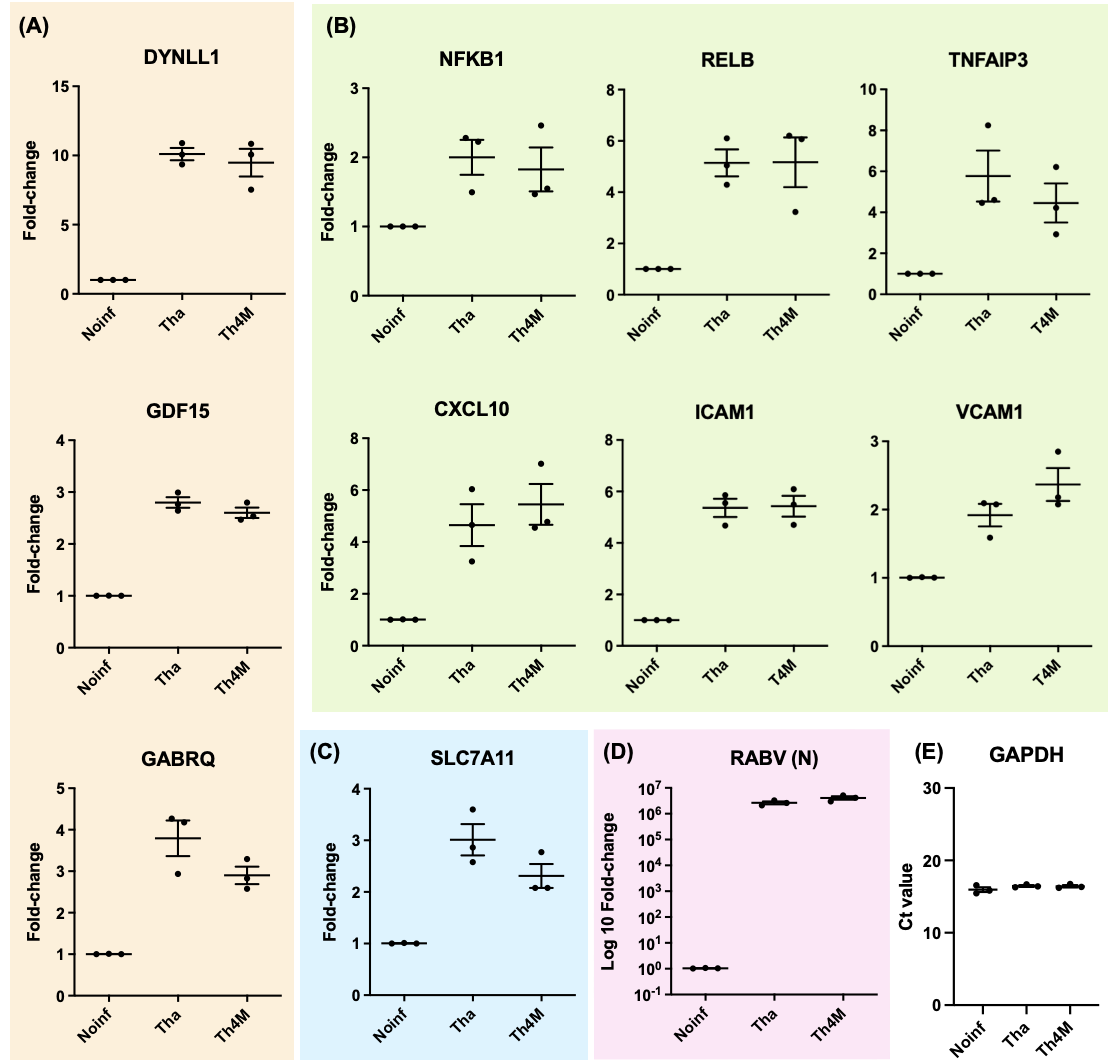


**Supplementary Figure 4.** RT-qPCR of neuronal function-related DEGs with RNAs prepared from H9 NSC-derived neurons. (A) DEGs associated with MAPK pathway, (B) DEGs with NF-kB pathway, (C) DEG with JAK-STAT pathway. (D) RABV RNA level was detected in RT-qPCR using N protein primers. (E) Reference gene GAPDH was detected at similar level of expression in all conditions.


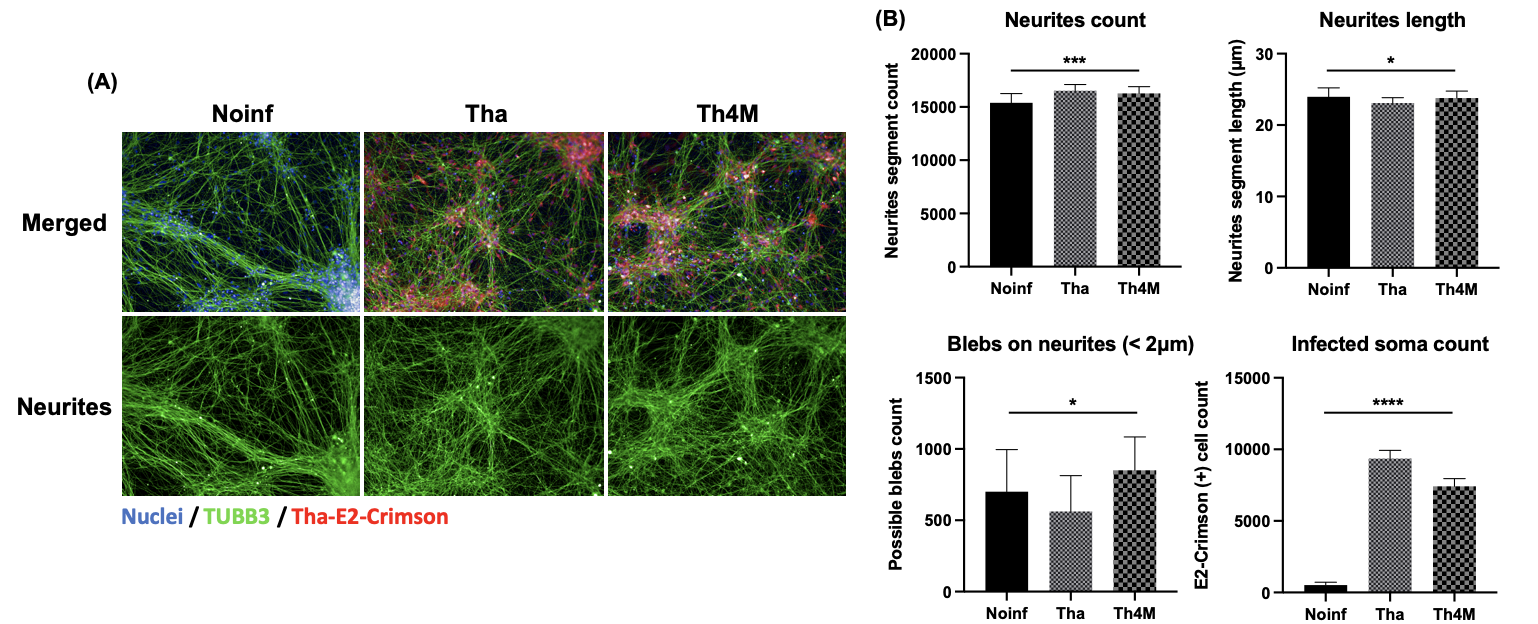


**Supplementary Figure 5.** Tha-infected neurons display higher number of neurites but less bleb structures on the neurites. (A) Images of mock, Tha- and Th4M-infected neurons, stained with TUBB3 antibody (neurites) and Hoechst 33342 (nuclei). (B) Measurement of features of neurite and signs of apoptosis, using Columbus Analysis System from PerkinElmer. One-way ANOVA was performed to measure statistical differences among the conditions.


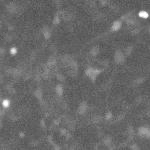


**Supplementary Figure 6.** An overview of the analysis workflow for calcium imaging. (A) Images of differentiated neurons were acquired in time-lapse (at 1.72 Hz for 4.5 minutes; Fluo-4 staining), and compiled in a video format to show that the activities of differentiated neurons are visible and detectable in all three experimental conditions (yellow circles; click on the images to play videos). (B) Motion correction crops the edge of images to align and find the commonly detected area in all frames. This is to remove motional artifacts that comes from
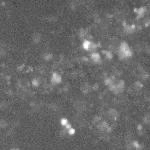

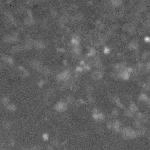

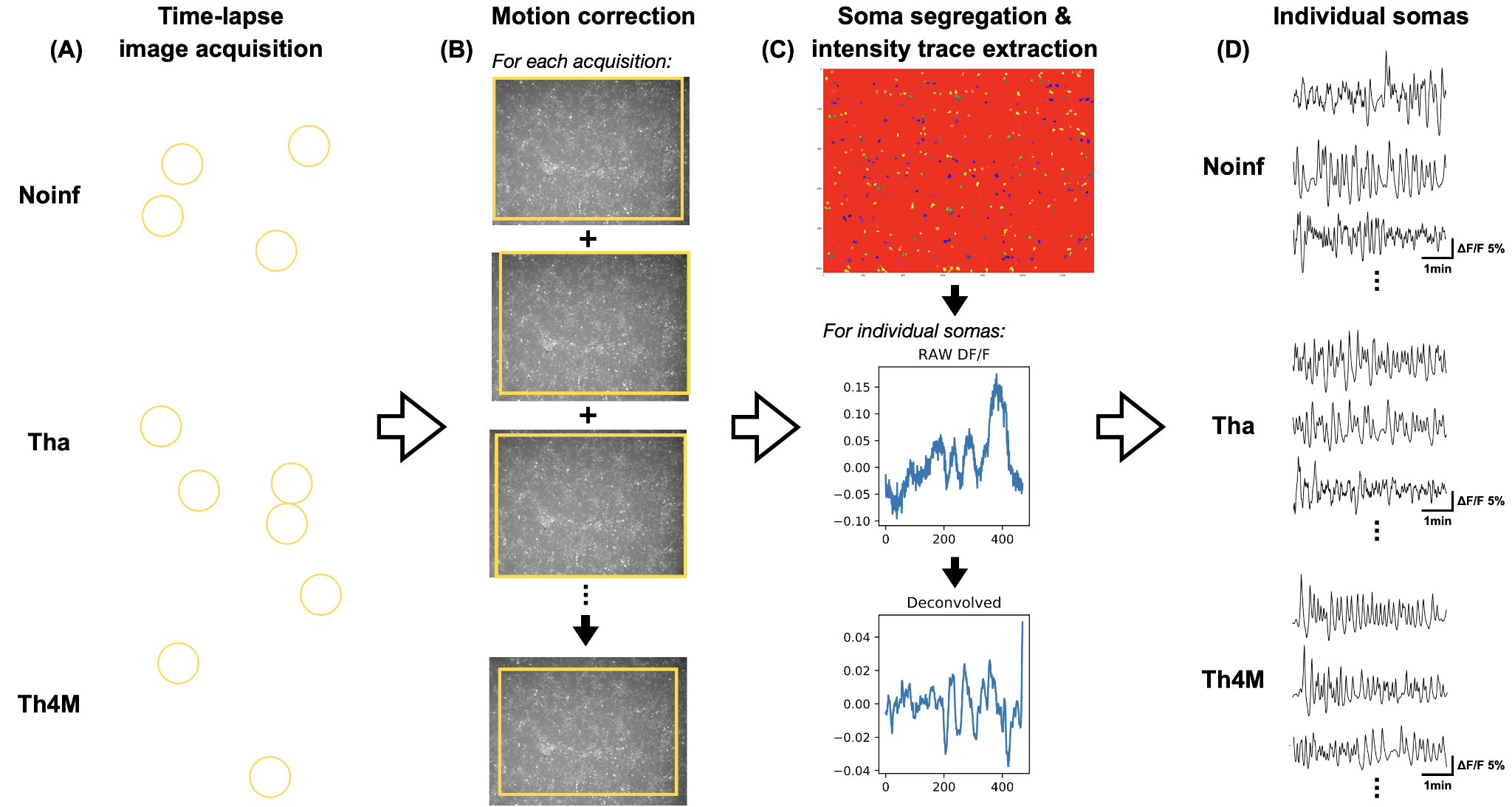
time-lapse imaging. (C) The regions for the individual somas were extracted from the corrected images, using a binary analysis (Independent Component analysis) and Watershed algorithm. Subsequently, the intensity trace (ΔF/F) of each soma was extracted and transformed to interpretable waveform, using a mathematical model of neuron impulse. (D) Representative intensity traces from active neurons in each experimental condition. From these traces, information on calcium peak frequencies and amplitude (height of calcium peak) was extracted for individual somas.
